# The mitotic checkpoint regulator RAE1 induces aggressive breast cancer cell phenotypes by mediating epithelial-mesenchymal transition

**DOI:** 10.1038/srep42256

**Published:** 2017-02-09

**Authors:** Ji Hoon Oh, Ho Hur, Ji-Yeon Lee, Yeejeong Kim, Younsoo Seo, Myoung Hee Kim

**Affiliations:** 1 Department of Anatomy, Embryology Laboratory, and Brain Korea 21 PLUS project for Medical Science, Yonsei University College of Medicine, Seoul 03722, Korea; 2 Department of Surgery, National Health Insurance Service Ilsan Hospital, Goyang 10444, Korea; 3 Department of Pathology, National Health Insurance Service Ilsan Hospital, Goyang 10444, Korea

## Abstract

The gene *RAE1* encodes ribonucleic acid export 1 (RAE1), which is involved in mRNA export and is known to serve as a mitotic checkpoint regulator. In addition, *RAE1* haplo-insufficiency leads to chromosome missegregation and early aging-associated phenotypes. In humans, a positive correlation has been found between *RAE1* copy number abnormalities and gene amplification in breast cancer cells. However, the precise functional role of RAE1 in breast cancer remains to be determined. An *in silico* analysis of data retrieved from GENT and cBio-Portal identified RAE1 upregulation in breast cancer tissues relative to normal breast cells. Functional studies of various cell lines showed that RAE1 induced invasive and migratory abilities by regulating epithelial-mesenchymal transition signals. A tissue microarray was constructed to demonstrate the interrelationship between clinicopathological features and RAE1 expression. Immunohistochemistry revealed a positive correlation between RAE1 expression and a high histologic grade. Furthermore, RAE1 overexpression was associated with considerably poorer disease-free survival and distant metastasis-free survival, especially in patients with oestrogen receptor-positive tumours. In summary, RAE1 may be a prognostic marker and therapeutic intervention target in malignant breast cancers.

Ribonucleic acid export 1 (Rae1) was originally discovered as an essential nucleocytoplasmic transport factor in yeast, and is now known to be involved in the export of nuclear mRNA to the cytoplasm^1^. A mammalian gene exhibiting homology to yeast *Rae1* has also been identified^2^; however, the products of these genes do not appear to share functional identities. Blastocysts of embryonic lethal Rae1-null mice exhibited no defects in nuclear pore complex (NPC) formation or the nuclear export of mRNA^3^. Rather, reports of *Rae1*-deficient models have described the involvement of Rae1 in chromosome missegregation or early aging, as well as chromosome instability and multi-spindle formation^3^,^4^. In contrast, the shuttling of human RAE1 between the nucleus and cytoplasm and the ability of RAE1 to form complexes with nuclear pore proteins suggest a role for RAE1 in mRNA export in humans^5^. These results demonstrate a critical role for mammalian RAE1 as a nuclear exporter of mRNA and/or mitotic checkpoint regulator to ensure the maintenance of proper spindle bipolarity^3^,^5^,^6^.

Aneuploidy and chromosome instability have long been proposed as contributors to tumour progression^7^,^8^,^9^. In addition, the association between NPC components and cancer has been well studied^10^. Accordingly, it is important to note that RAE1 might contribute to oncogenesis and cancer progression. RAE1 is known to be involved in nucleoporin 98 kDa (NUP98)-mediated tumorigenesis in acute myeloid leukaemia^11^,^12^, and aberrant RAE1 overexpression has been reported in lung cancer^13^. Regarding breast cancer, RAE1 is associated with genome amplification and copy-number driven expression and has thus been proposed as a putative oncogene^14^. Furthermore, genomic and transcriptional *RAE1* aberrations were found to be associated with a reduced survival duration among patients with breast cancer^15^. However, the exact roles of RAE1 and related abnormalities in breast cancer remain unclear.

In this study, which aimed to explore the relationship between RAE1 expression and breast cancer progression, we performed functional studies of breast cancer cell lines and analysed the relationships of RAE1 expression with clinicopathological features and prognosis in patients with breast cancer. Through RAE1 overexpression and knockdown studies, we revealed that RAE1 enhanced aggressive breast cancer cell phenotypes by inducing epithelial-mesenchymal transition (EMT) signals. A combined tissue microarray (TMA) and survival analysis revealed the prognostic significance of RAE1 and a positive correlation between RAE1 expression and histologic grade in invasive ductal carcinomas.

## Results

### RAE1 abnormalities in breast cancer

**Figure 1:**
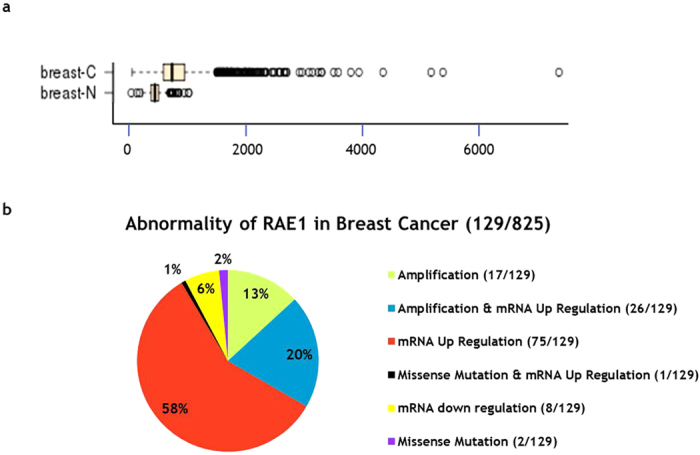
*RAE1* abnormalities in human breast cancers. (**a**) Public data retrieved from GENT (http://medical-genome.kribb.re.kr/GENT) indicate that RAE1 is upregulated in breast cancer tissues. The GSE2019 data set was used for this analysis. Each circle represents an individual tissue sample. The X-axis of the plot indicates normalized expression measures. Significance was analysed using an unpaired t-test (*p* = 2.61087E-45, breast-N: n = 271, breast-C: n = 2,658). (**b**) *RAE1* abnormalities in breast cancers according to cBioPortal data (http://www.cBioPortal.org).

To investigate the relationship between *RAE1* expression and breast cancer, we analysed *in silico* data from the Gene Expression across Normal and Tumour tissue database (GENT; http://medical-genome.kribb.re.kr/GENT). A comparison of 271 normal breast tissues with 2,658 breast cancer tissues clearly demonstrated significant upregulation of RAE1 in the latter (Fig. 1a). An analysis of retrieved data from cBio-Portal (http://www.cbioportal.org) specified the classes of these abnormalities. Among 825 evaluated patients with breast cancer, 16% (n = 129) harboured abnormalities in *RAE1*, including amplification, mRNA up/downregulation, and missense mutations. Of those abnormalities, mRNA upregulation was the most common (120/129, 79%; Fig. 1b). These data demonstrate that RAE1 upregulation might associate with breast cancer development.

### Effects of RAE1 overexpression and knockdown in breast cancer cell lines

Given the above results, which indicate the significance of RAE1 overexpression in breast cancer, we investigated the function of RAE1 in various breast cancer cell lines, including MCF7 (oestrogen receptor [ER]-positive), T47D (ER-positive), and MDA-MB-231 (triple negative). Each cell line was transfected with the pCMV6-RAE1 plasmid and subjected to a 2–3 week G418 selection period to generate stable RAE1-overexpressing lines (MCF7:RAE1 #1, 2, 3; T47D:RAE1 #1, 2, 3; MDAMB231:RAE1 #1, 2, 3). As controls, we used stable cell lines transfected with empty vectors (MCF7:empty vec #1, 2; T47D:empty vec #1, 2; MDAMB231:empty vec #1, 2).

**Figure 2:**
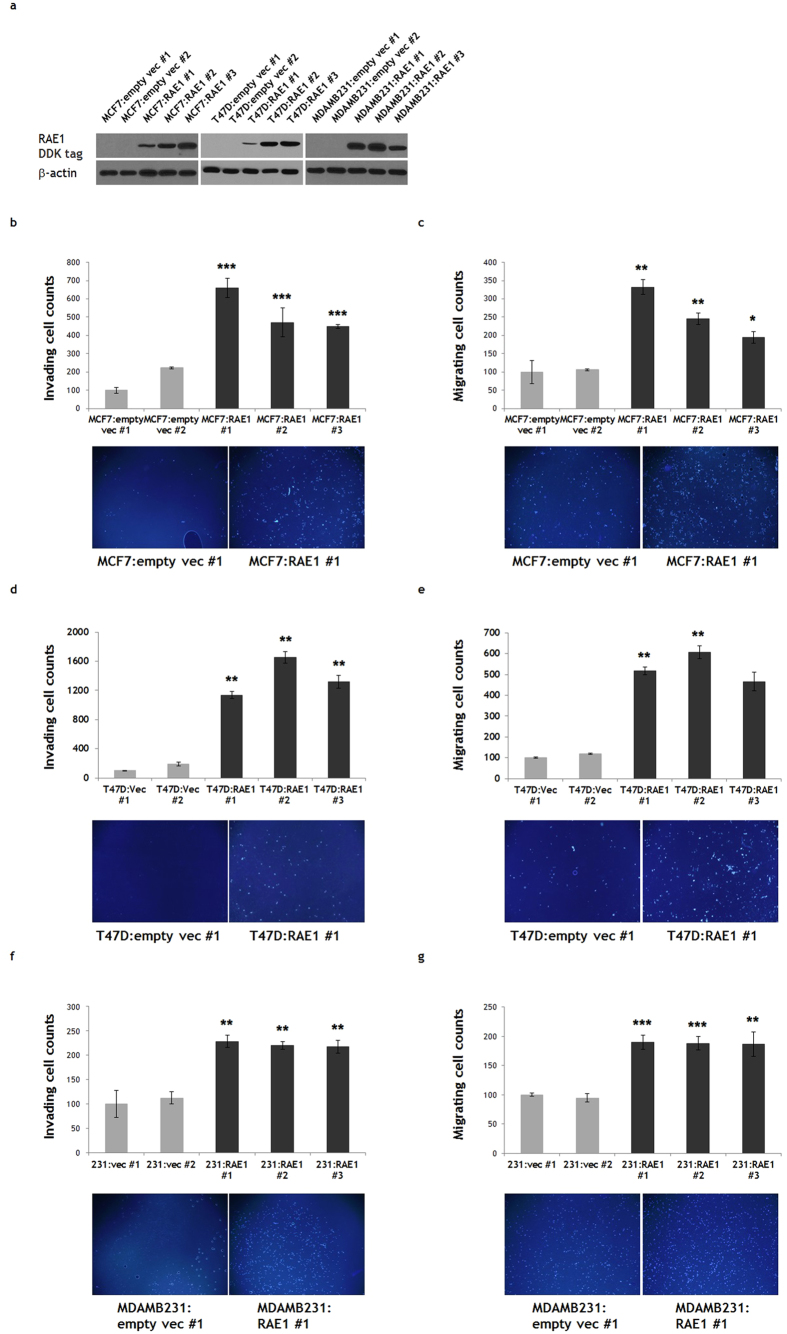
Effect of RAE1 overexpression in breast cancer cell lines. (**a**) Western blotting results showing stable overexpression of RAE1 in the MCF7, T47D, and MDA-MB-231 breast cancer cell lines. An anti-DDK antibody was used to detect overexpressed RAE1. Full-length blots are presented in Supplementary Fig. 1. (**b**–**g**) A Matrigel invasion assay and migration assay were performed to measure the invasive (**b**,**d**,**f**) and migratory (**c**,**e**,**g**) abilities, respectively, of MCF7, T47D, and MDA-MB-231 cells. *p < 0.05, **p < 0.01, ***p < 0.001.

RAE1 overexpression was confirmed by western blotting with an anti-DDK antibody (Fig. 2a). Control and RAE1-overexpressing cells did not differ significantly in terms of proliferative activities or apoptosis (data not shown), suggesting that RAE1 does not influence breast cancer cell survival. Rather, RAE1-overexpressing cell populations included higher numbers of invading/migrating cells relative to controls, regardless of cell type (Fig. 2b–g). These data indicate that RAE1 overexpression affects breast cancer cell aggressiveness by inducing migratory and invasive abilities.

**Figure 3:**
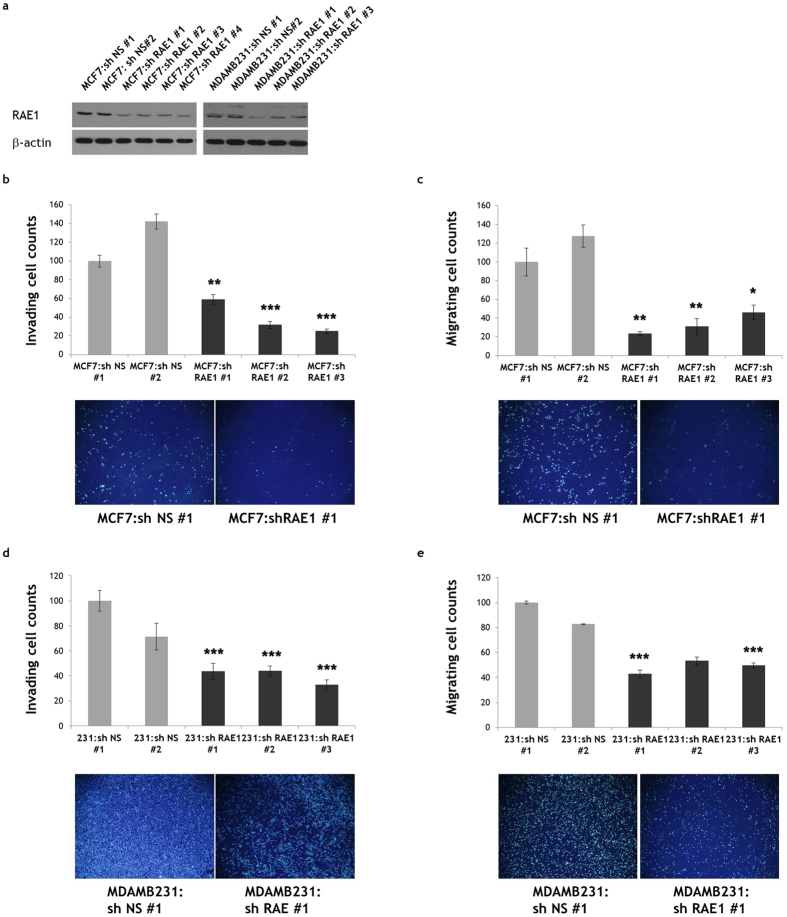
Effect of RAE1 knockdown in breast cancer cell lines. (**a**) Western blot results showing the stable knockdown of RAE1 in MCF7 and MDA-MB-231 breast cancer cell lines. An anti-RAE1 antibody was used to detect RAE1. Full-length blots are presented in Supplementary Fig. 2. (**b**–**e**) A Matrigel invasion assay and migration assay were performed to measure the invasive (**b**,**d**) and migratory (**c**,**e**) abilities, respectively, of MCF7 and MDA-MB-231 cells. *p < 0.05, **p < 0.01, ***p < 0.001.

To investigate whether RAE1 deficiency would induce the opposite effects in breast cancer cells, we generated stable RAE1-knockdown MCF7 and MDA-MB-231 cells (MCF7:shRAE1 #1, 2, 3, 4 and MDAMB231:shRAE1 #1, 2, 3) and control cell lines (MCF7:sh NS #1, 2 and MDAMB231:sh NS #1, 2) after infection with lentiviral particles containing *RAE1* shRNA and selection. RAE1 protein downregulation in each stable RAE1*-*knockdown cell line was confirmed by western blotting with an anti-RAE1 antibody (Fig. 3a). As expected, RAE1 downregulation reduced the invasive and migratory capacities of breast cancer cells (Fig. 3b–e), implicating a possible role of RAE1 in breast tumour cell invasion and metastasis.

### The relationship between RAE1 and EMT

**Figure 4:**
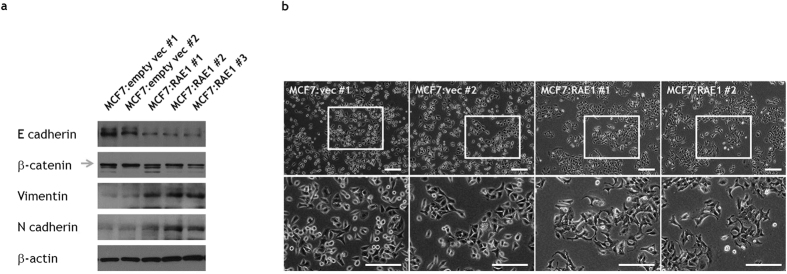
Effect of RAE1 overexpression or knockdown in breast cancer cell lines. (**a**) Immunoblot analysis of E-cadherin, β-catenin, vimentin, and N-cadherin expression in stable RAE1-overexpressing MCF7 cells. Full-length blots are presented in Supplementary Fig. 3. (**b**) A morphological change from a cobblestone shape to an elongated spindle shape was observed in MCF7 cells after inducing RAE1 overexpression. Scale bar = 200 μm.

After observing that RAE1 induced migratory and invasive abilities in breast cancer cells in our *in vitro* system, we tested whether the EMT mediated this phenomenon. To evaluate changes in EMT-related proteins, we performed western blotting for major epithelial (E-cadherin and β-catenin) and mesenchymal markers (vimentin and N-cadherin) in RAE1-overexpressing cells. Notably, epithelial markers were downregulated, whereas mesenchymal markers were upregulated (Fig. 4a). In contrast, the protein levels of E-cadherin and β-catenin were upregulated in RAE1-knockdown cells (Fig. S4). Interestingly, RAE1 overexpression in MCF7 cells induced a morphological change from a normal fibroblast spindle cell shape to a cobblestone-like shape (Fig. 4b). Similar morphological changes were also detected in RAE1-overexpressing T47D cells (Fig. S5a). The opposite morphological pattern was observed in RAE1-knockdown MDA-MB-231 cells (Fig. S5b). Taken together, our *in vitro* data suggest that RAE1 induces EMT and thus promotes the migration and invasion of breast cancer cells.

### Correlation of RAE1 protein expression with clinicopathological features and prognosis in patients with breast cancer

**Figure 5:**
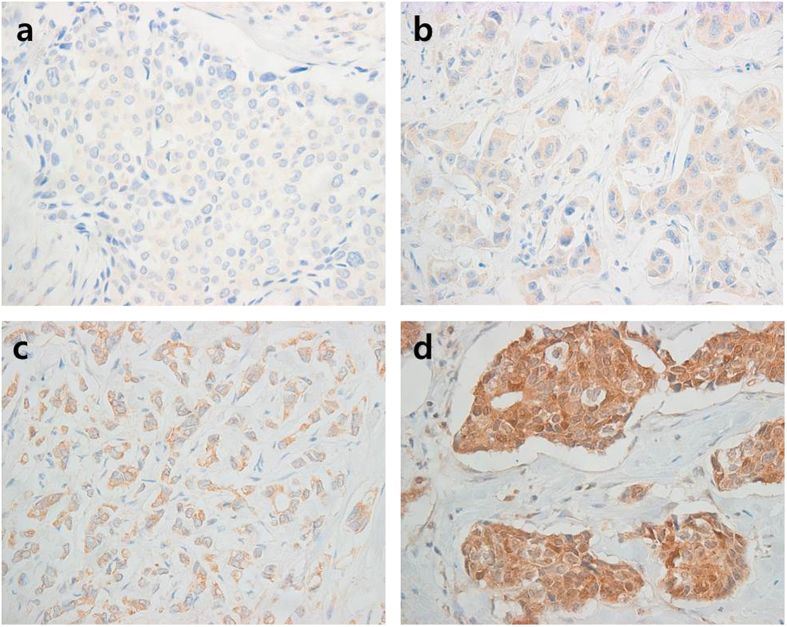
Immunohistochemical staining for RAE1. RAE1 expression levels are categorized as negative (**a**), weak (**b**), moderate (**c**), or strong (**d**) according to the RAE1 immunostaining signal intensity score (magnification, 400 ×).

As RAE1 induces EMT and invasion, we examined the potential correlations of clinicopathological features with RAE1 expression in patients with breast cancer. We used breast cancer tissues from 98 patients to construct a TMA, which was subjected to immunohistochemistry (IHC) to evaluate RAE1 protein expression (Fig. 5). Breast cancer tissues with an Allred score of ≥3 are typically considered positive. In our samples, although 25.5% (25/98) of breast cancer tissues were defined as RAE1-positive (Table 1), we were unable to find any correlations with various clinicopathological features. However, using an Allred score of 5 as a cut-off value, we observed a positive correlation between RAE1 and a high histologic grade in 16.3% of tissues (16/98) with strong signals (Allred score ≥5; Table 2). However, the prognostic impact of RAE1 overexpression could not be determined because of the low number of positive cases in our dataset.

**Figure 6:**
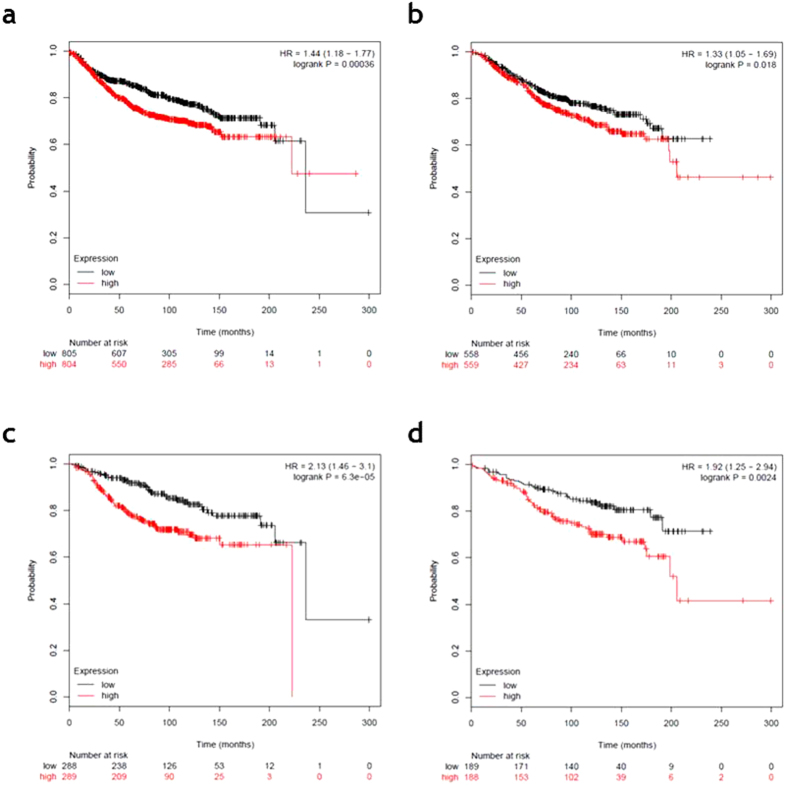
Kaplan–Meier analysis of distant metastasis-free survival (DMFS) and overall survival (OS) according to online dataset records of *RAE1* expression. Survival was compared between the *RAE1*-high and -low expression groups. (**a**) DMFS (n = 1609, number of events = 804, *p* = 0.00036) and (**b**) OS (n = 1117, number of events = 559, *p* = 0.018) for all breast cancer patients in the dataset. (**c**) DMFS (n = 577, number of events = 289, *p* = 6.3e-05) and (d) OS (n = 377, number of events = 188, *p* = 0.0024) for oestrogen receptor (ER)-positive breast cancer patients in the dataset.

Therefore, we examined the effect of RAE1 expression on the prognosis of breast cancer patients using a Kaplan–Meier online tool (http://kmplot.com). Among all patients, higher RAE1 expression was associated with worse distant metastasis-free survival (DMFS; *p* = 0.00036; Fig. 6a) and overall survival (OS; *p* = 0.018; Fig. 6b). Among ER-positive patients, high RAE1 expression associated strongly with a poor DMFS (*p* = 6.3e-05; Fig. 6c) and OS (*p* = 0.0024; Fig. 6d).

## Discussion

This is the first study to investigate in depth the functional roles and clinical significance of RAE1 in breast cancer using both *in vitro* system and patient samples, together with an *in silico* analysis. Our integration of *in silico* resources confirmed the overexpression of *RAE1* in patients with breast cancer, in accordance with previous reports that demonstrated *RAE1* copy number variation (CNV) and gene expression abnormalities^14^,^15^. In addition, our confirmation of RAE1-positive patients among a larger population of patients with breast cancer and the relationship of this parameter with clinicopathological data via TMA analysis provides worthwhile information. Our important findings, namely the correlations of RAE1 overexpression with a high histologic grade and poor prognosis and identification of a functional role of RAE1 in cancer cell migration and invasion, strongly support the notion that RAE1 contributes to breast cancer progression.

Our *in vitro* experiment demonstrated that the expression of EMT markers, such as E-cadherin, vimentin, and N-cadherin, was modulated by RAE1 expression and, moreover, that the phenotypes of various cell lines shifted between epithelial and mesenchymal states depending on RAE1 expression levels. Because EMT enhances the metastatic potential of breast cancer cells, a critical determinant of the prognosis of a patient with breast cancer^16^, our results strengthen the relationship between RAE1 activity and breast cancer aggressiveness. The histologic grades of invasive breast cancers are assigned based on the structures of cancer cells. A high histologic grade indicates a poorly differentiated tumour with insufficient tubule formation. Given the role of E-cadherin in the maintenance of duct formation and epithelial integrity^17^,^18^,^19^, our data provide a plausible explanation for the *in vivo* function of RAE1; specifically, strong RAE1 expression may facilitate an EMT-like switch and act as an unfavourable metastatic factor, leading to an invasive ductal histology and high histological grade.

In addition to the impact of RAE1 gene overexpression, dysregulation of RAE1 can lead to chromosome missegregation^3^, chromosomal instability, and multipolar spindles, with consequent aneuploidy^6^,^20^,^21^,^22^. As chromosome number alterations, a consequence of chromosome missegregation, are a known hallmark of cancer cells^7^,^8^,^9^,^23^, RAE1 deficiency or loss could contribute to cancer development or progression. However, the low incidence of RAE1 mRNA downregulation or missense mutation events in human breast cancers (Fig. 1b) suggests that RAE1 deficiency might not be a major driving force in this type of cancer. Instead, *RAE1* gene amplification and consequent RAE1 overexpression appear to be important risk factors in breast cancers. Recently, studies that have identified and evaluated the functional consequences of CNV loci have received attention in the field of cancer biology^15^,^24^,^25^,^26^. Similar to RAE1, amplification and overexpression of BUB3, a mitotic checkpoint regulator that shares extensive sequence homology with RAE1^3^, were found to correlate with the luminal A subtype of breast cancer^26^. Chromosome loci 8p11-12, 11q13-14, and 20q13 in which *RAE1* is located, have been identified as amplification hotspots that correlated with poor survival in a group of patients with luminal A subtype breast cancer^15^. Furthermore, the functional significances of several genes, such as *LSM1, TACC1, ADAM9, IKBKB, POLB*, and *FGFR1* at 8p11 and *CCND1* and *FGF3* at 11q13, have been identified^15^. In particular, *ZNF217*, which is located on 20q13 near the locus that encodes RAE1, was previously reported as a novel breast cancer gene along with *EGFR1, ERBB2,* and *PPMID*^26^. Among the related factors, FGFR1, IKBKB, and ERBB2 have been targeted for therapeutic drug development (PD173074 against FGFR1, PS-1145 against IKBKB, and trastuzumab against ERBB2)^26^.

In conclusion, our results, which demonstrate the functional role of RAE1 in cancer cell migration and invasion via EMT involvement, imply an important role for RAE1 during specific stages of cancer progression and metastasis. Given the findings regarding the relationship between RAE1 overexpression and clinicopathological significance in breast cancer, RAE1 might represent a new therapeutic target and prognostic marker.

## Methods

### Cell culture, plasmids, and generation of stable cell lines

MCF7, T47D, and MDA-MB-231 cells were cultured as previously described^27^. For overexpression studies, full-length *RAE1* cDNA was cloned in a pCMV6 expression vector containing the Myc-DDK-tag (#PS100001; Origene, Rockville, MD, USA); this vector construct was subsequently transfected into MCF7, T47D, and MDA-MB-231 cells using Attractene reagent (Qiagen, Venlo, Netherlands). For controls, cells were transfected with an empty pCMV6 vector. Transfected cells were treated with G418 (500 μg/ml; Gibco, Grand Island, NY, USA) for 2–3 weeks to generate stable cell lines. For knockdown experiments, MCF7 and MDA-MB-231 cells were transduced with pGFP-C-shLenti virus particles specific for *RAE1* (#TR30023; Origene) or control shRNA. These cells were treated with puromycin (0.5 μg/ml; Sigma, St. Louis, MO, USA) for 7 days to generate stable cell lines.

### Western blotting

Western blot analyses were performed as previously described^27^. Anti-RAE1 (Abcam, Cambridge, UK), anti-E-cadherin (Abcam), anti-β-catenin (BD Biosciences, San Diego, CA, USA), anti-DDK-tag mouse monoclonal antibody (Origene), anti-N-cadherin (Abcam, Cambridge, UK), anti-vimentin (Sigma), and anti-β-actin (Sigma) were used to detect each protein.

### Matrigel invasion and migration assays

The Matrigel™ (BD, Franklin Lakes, NJ, USA) invasion assay was performed as previously described^27^. The migration assay was performed using the same protocol in the absence of Matrigel™. For these experiments, 5 × 10^4^ cells were placed in each chamber. The same number of cells was used regardless of cell line. After a cell line-dependent 6–72-hour incubation, invading cells were stained with fluorochrome 4′,6-diamidino-2-phenylindole (DAPI) and observed via fluorescent microscopy. Acquired images were analysed using ImageJ software (National Institutes of Health, Bethesda, MD, USA).

### Tissue microarray and immunohistochemistry (TMA-IHC)

From among patients who underwent surgical resection of breast cancer between January 2006 and December 2010 at the National Health Insurance Service Ilsan Hospital, Gyeonggi-do, Korea, 103 patients with well-preserved paraffin-embedded tissue blocks were selected because sufficient tumour for TMA construction was available in a single tissue block. Data concerning the primary tumour histopathology and patient characteristics were retrospectively obtained by reviewing medical records; five patients who received preoperative chemotherapy were subsequently excluded. The Ethics Committee for the Clinical Research of the Institutional Review Board of the National Health Insurance Service Ilsan Hospital, Gyeonggi-do, Korea, approved this study protocol (2016-07-016). All tissue microarray experiments were performed in accordance with the guidelines and regulations set by the ethics committee. Furthermore, all patient samples were collected after obtaining informed consent. TMA construction and IHC analysis were performed as previously described^27^. For IHC, a primary antibody against RAE1 (Abcam) was used. Immunostaining signals were scored using the Allred scoring system.

### *In silico* analysis

The Gene Expression across Normal and Tumour Tissue (GENT) web-accessible database (http://medical-genome.kribb.re.kr/GENT) was used to evaluate *RAE1* expression patterns in breast cancer tissues. The web-accessible database cBioPortal (http://www.cbioportal.org) was also used to evaluate *RAE1* abnormalities in breast cancer tissues. A Kaplan–Meier plotter (http://kmplot.com) was used for the survival analysis.

### Statistical analysis

Clinicopathological variables were compared using the chi-square test or Fisher’s exact test. All statistical tests were two-sided, and *p*-values of <0.05 were considered statistically significant. SPSS for Windows, version 23.0 (SPSS Inc., Chicago, IL, USA) was used for the statistical analyses.

## Additional Information

**How to cite this article**: Oh, J. H. *et al*. The mitotic checkpoint regulator RAE1 induces aggressive breast cancer cell phenotypes by mediating epithelial-mesenchymal transition. *Sci. Rep.* **7**, 42256; doi: 10.1038/srep42256 (2017).

**Publisher's note:** Springer Nature remains neutral with regard to jurisdictional claims in published maps and institutional affiliations.

## Supplementary Material

Supplementary Information

## Figures and Tables

**Table 1:** RAE1 expression in breast cancer specimens.

	RAE1 negative (n = 73)		RAE1 positive (n = 25)					
Allred score	0	2	3	4	5	6	7	8
Cases	71	2	3	6	3	8	5	0

**Table 2:** Correlation analysis between RAE1 and clinicopathological features.

	Allred score 3			Allred score 5		
	<3	≥3	*p*-value	<5	≥5	*p*-value
	n = 73	n = 25		n = 82	n = 16	
Tumour size			0.397			0.794
≤2 cm	45 (61.6%)	13 (52.0%)		49 (59.8%)	9 (56.3%)	
>2 cm	28 (38.4%)	12 (48.0%)		33 (40.2%)	7 (43.8%)	
Lymph node			0.537			1.000
Negative	41 (56.9%)	16 (64.0%)		48 (59.3%)	9 (56.3%)	
Positive	31 (43.1%)	9 (36.0%)		33 (40.7%)	7 (43.8%)	
Unknown				1	0	
Histologic grade			0.187			**0.013**
I, II	46 (63.0%)	12 (48.0%)		53 (64.6%)	5 (31.3%)	
III	27 (37.0%)	13 (52.0%)		29 (35.4%)	11 (68.8%)	
Oestrogen receptor			0.947			0.343
Negative	18 (24.7%)	6 (24.0%)		22 (26.8%)	2 (12.5%)	
Positive	55 (75.3%)	19 (76.0%)		60 (73.2%)	14 (87.5%)	
Progesterone receptor			0.469			0.865
Negative	29 (39.7%)	12 (48.0%)		34 (41.5%)	7 (43.8%)	
Positive	44 (60.3%)	13 (52.0%)		48 (58.5%)	9 (56.3%)	
HER2			0.729			0.548
Negative	46 (63.9%)	15 (60.0%)		52 (64.2%)	9 (56.3%)	
Positive	26 (36.1%)	10 (40.0%)		29 (35.8%)	7 (43.8%)	

HER2, human epidermal growth factor receptor 2.
