# Supplementary material for: The mitotic checkpoint regulator RAE1 induces aggressive breast cancer cell phenotypes by mediating epithelial-mesenchymal transition
Source: Sci Rep. 2017 Feb 9;7:42256. doi: 10.1038/srep42256 (PMC5299842; doi:10.1038/srep42256)

Supplementary Information

The mitotic checkpoint regulator RAE1 induces aggressive breast cancer cell phenotypes by mediating epithelial-mesenchymal transition

Ji Hoon Oh[1†], Ho Hur[2†], Ji-Yeon Lee[1], Yeejeong Kim[3], Youn Soo Seo[1], Myoung Hee Kim[1*]

Figure S1. RAE1 overexpression in breast cancer cell lines. Full-length western blot results showing stable overexpression of RAE1 in (a) MCF7, (b) T47D, and (c) MDA-MB-231 breast cancer cell lines. (a) R2, R5, and R11 were the selected RAE1 overexpressing MCF7 colonies, and were labelled in the main figures to be MCF7:RAE1 #1, 2, 3. (b) R2, R5, and R7 were the selected RAE1 overexpressing T47D colonies, and were labelled in the main figures to be T47D:RAE1 #1, 2, 3. (c) R1, R2, and R8 were the selected RAE1 overexpressing MDA-MB-231 colonies, and were labelled in the main figures to be MDAMB231:RAE1 #1, 2, 3. An anti-DDK antibody was used to detect overexpressed RAE1. An anti-β-actin antibody was used as a control. β-actin (42 kDa) could not be run on the same blot, because it has a similar molecular weight to RAE1 (41 kDa). Same cell lysates were used for β-actin and RAE1.

Figure S2. RAE1 knockdown in breast cancer cell lines. Full-length western blot results showing stable knockdown of RAE1 in MCF7 and MDA-MB-231 breast cancer cell lines. sh1, 2, 3, and 4 were the selected RAE1 knockdown MCF7 colonies, and were labelled in the main figures to be MCF7:shRAE1 #1, 2, 3, 4. sh1, 2, and 3 were the selected RAE1 knockdown MDA-MB-231 colonies, and were labelled in the main figures to be MDAMB231:shRAE1 #1, 2, 3. An anti-RAE1 antibody was used to detect RAE1. An anti-β-actin antibody was used as a control. β-actin (42 kDa) could not be run on the same blot, because it has a similar molecular weight to RAE1 (41 kDa). Same cell lysates were used for β-actin and RAE1.

Figure S3. Effect of RAE1 overexpression in MCF7 cells. Immunoblot analysis of E-cadherin, β-catenin, Vimentin and N-cadherin in stable RAE1-overexpressing MCF7 cells. For the detection of E-cadherin and β-catenin, 40 ug of cell lysates were used. In addition, 30

ug of cell lysates were used for detection of Vimentin and N-cadherin. However, β-actin detection using the same amount of cell lysates resulted in over-saturation. Therefore, β-actin was run on a different blot, using 5 ug of cell lysates. The unlabelled bands are irrelevant to this study, and can be disregarded.

Figure S4. Effect of RAE1 knockdown in MCF7 cells. (a) Immunoblot analysis of E-cadherin and β-catenin in stable RAE1-knockdown MCF7 cells. For the detection of E-cadherin and β-catenin, 40 ug of cell lysates were used. However, β-actin detection using the same amount of cell lysates resulted in over-saturation. Therefore, β-actin was run on a different blot, using 5 ug of cell lysates. The unlabelled bands are irrelevant to this study, and can be disregarded. (b) Full-length blots of E-cadherin, β-catenin and β-actin in stable RAE1-knockdown MCF7 cells.

Figure S5. A morphological change of breast cancer cells following the dysregulation of RAE1. A morphological change from a cobblestone shape to an elongated spindle shape was observed in (a) T47D cells after RAE1 overexpression. The opposite pattern was observed in the RAE1-knockdown (b) MDA-MB-231 cells. The lower panel shows a 2.5 x magnified image of the squared portion of the upper panel. Scale bar = 200 um.

Supplementary Fig. S1

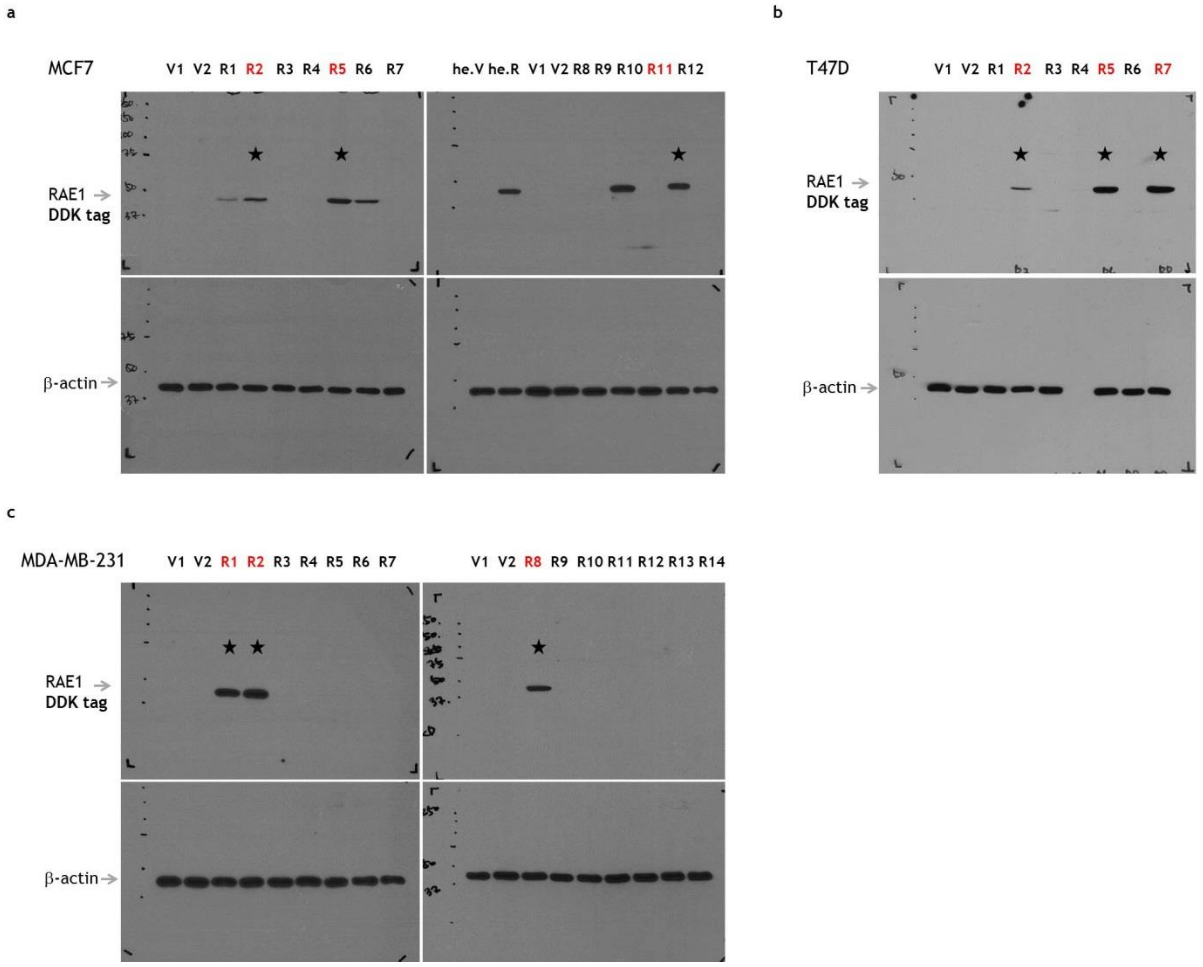

Supplementary Fig. S2

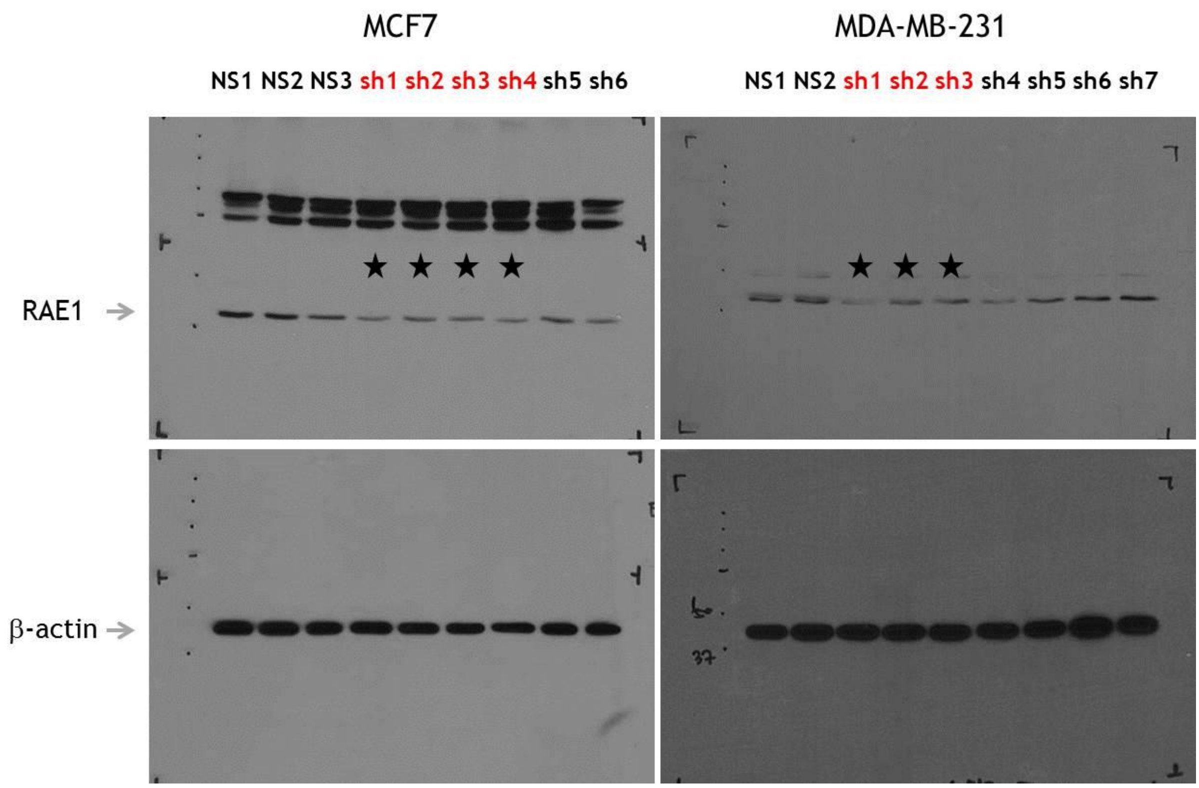

Supplementary Fig. S3

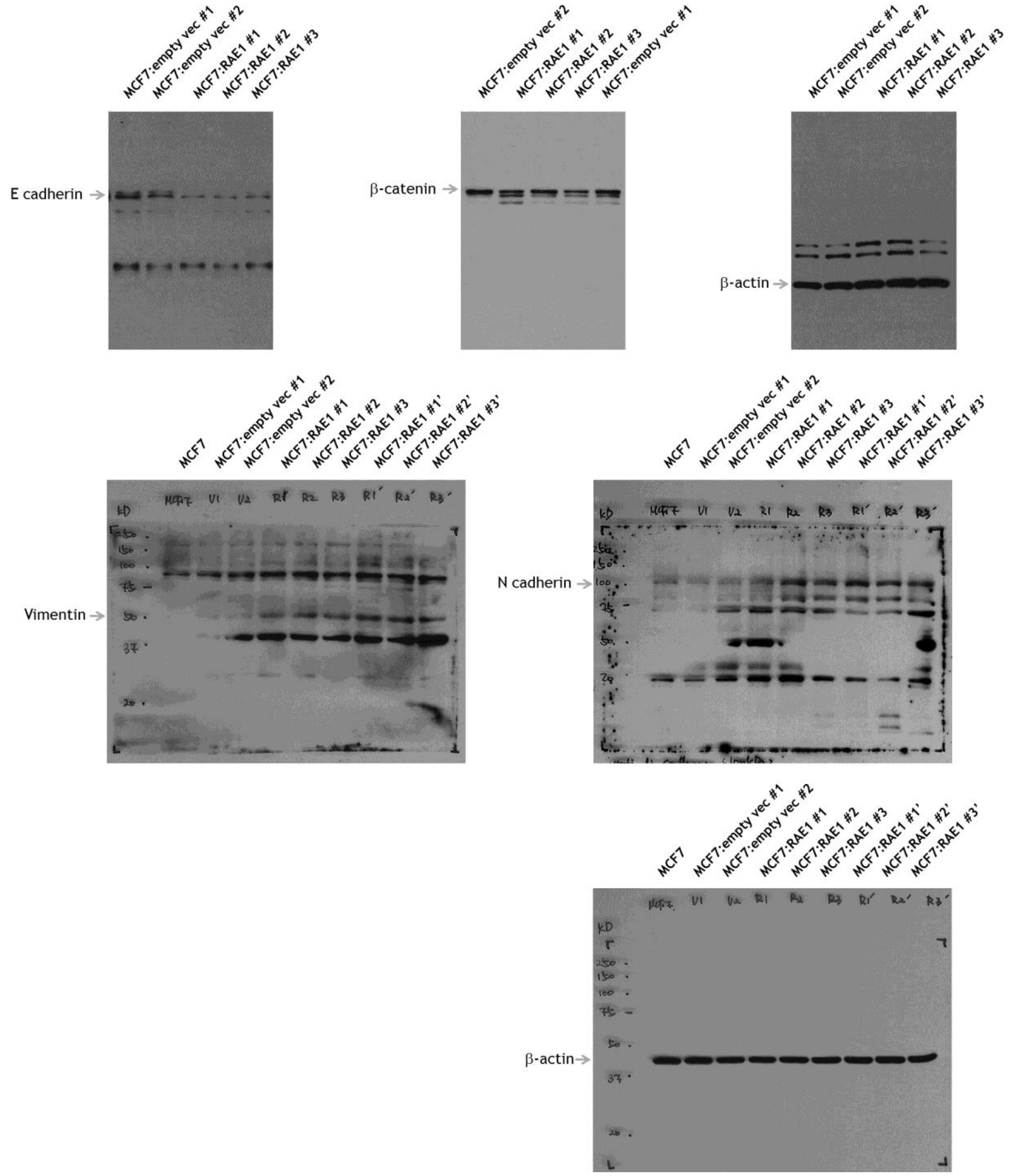

Supplementary Fig. S4

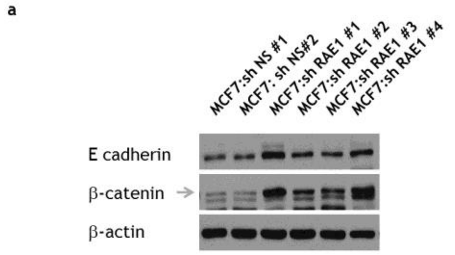

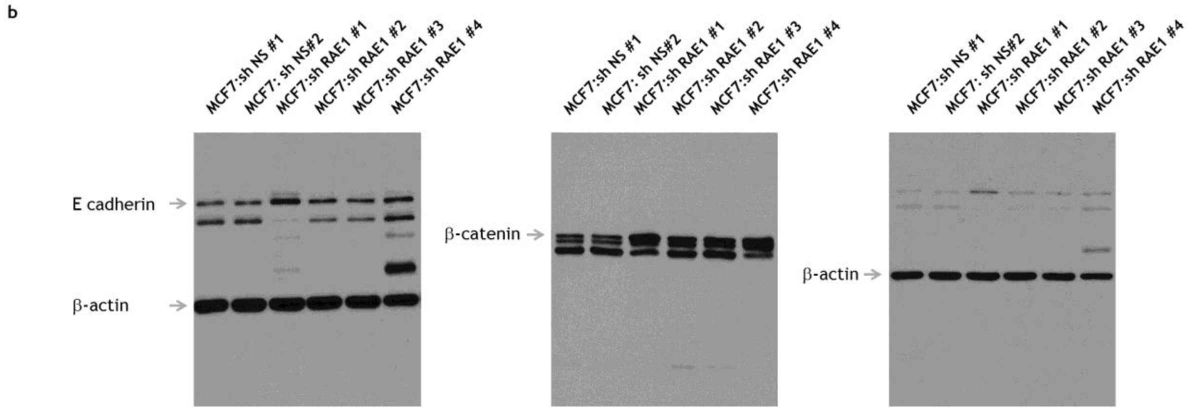

Supplementary Fig. S5

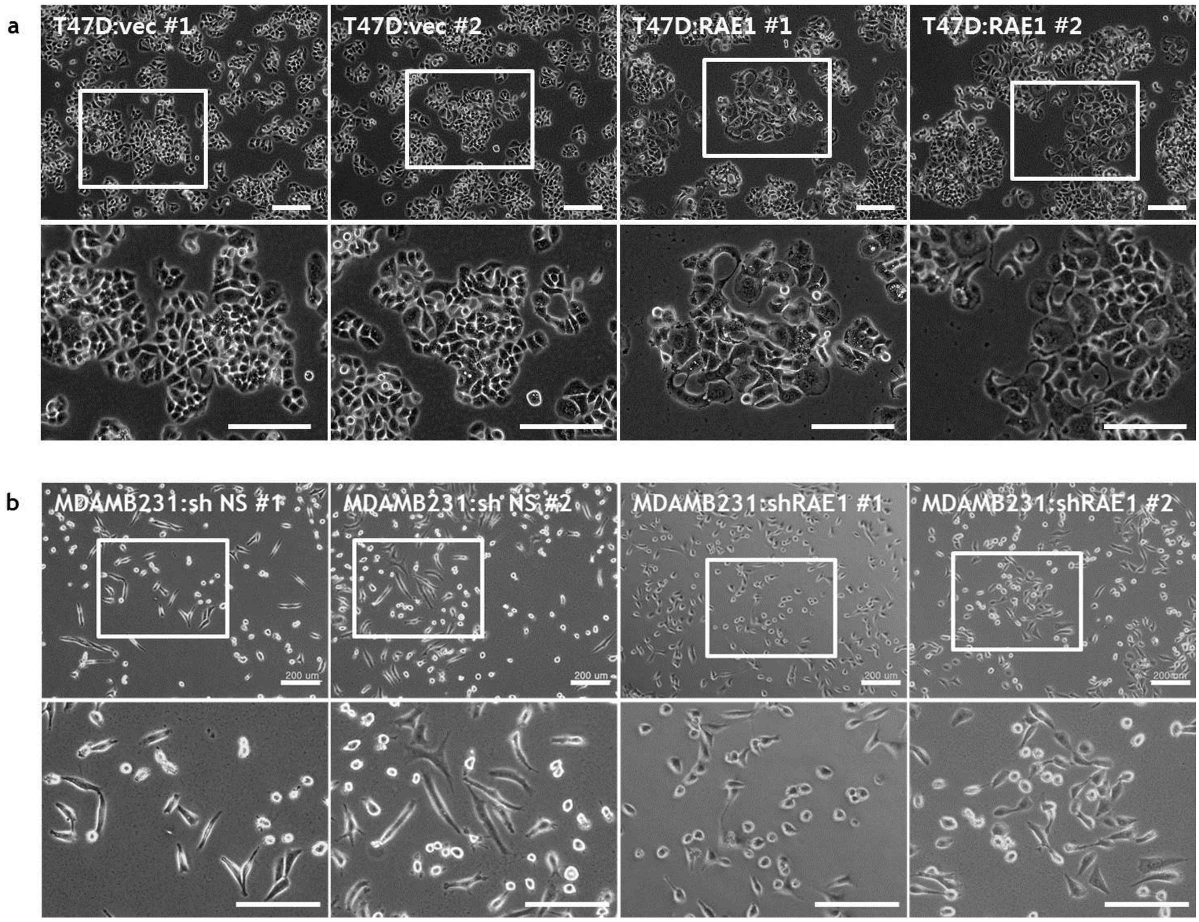

Supplement: Supplementary Information [file srep42256-s1.pdf]
